# Supplementary material for: Nested Real-Time PCR Assessment of Vertical Transmission of Sandalwood Spike Phytoplasma (‘Ca. Phytoplasma asteris’)
Source: Biology (Basel). 2022 Oct 12;11(10):1494. doi: 10.3390/biology11101494 (PMC9598993; doi:10.3390/biology11101494)
Supplement: Supplementary file 1 [file biology-11-01494-s001.zip › biology-1874056-supplementary.pdf]

Supplementary data

Table S1. The top-hit taxon of endophytic bacterial strains isolated from Symptomatic seedlings (30-40 DAS) that tested negative for phytoplasma.

Strain ID	Top-hit taxon (EzBiocloud database)	Similarity (%)	Completeness (%)	GenBank Acc No.
SR01	*Bacillus aryabhattai* B8W22	100	100	OM838450
SR07	*Bacillus aryabhattai* B8W22	100	100	OM838453
SRD03	*Bacillus aryabhattai* B8W22	99.86	100	OM838455
ST05	*Bacillus aryabhattai* B8W22	99.93	100	OM838466
STD02	*Bacillus aryabhattai* B8W22	100	100	OM838468
STD03	*Bacillus aryabhattai* B8W22	100	100	OM838469
STD01	*Bacillus cereus* ATCC 14579	100	100	OM838467
SRD10	*Bacillus filamentosus* SGD-14	100	100	OM838462
SR05	*Bacillus megaterium* NBRC 15308	100	100	OM838452
SRD04	*Bacillus megaterium* NBRC 15308	100	100	OM838456
ST04	*Bacillus megaterium* NBRC 15308	100	100	OM838465
ST02	*Bacillus mobilis* 0711P9-1	99.93	100	OM838464
SRD01	*Bacillus tequilensis* KCTC 13622	99.39	100	OM838454
SR02	*Bacillus velezensis* CR-502	99.86	99.9	OM838451
SRD05	*Curtobacterium oceanosedimentum* ATCC 31317	99.3	98.8	OM838457
STD04	*Curtobacterium oceanosedimentum* ATCC 31317	99.51	98.5	OM838470
AR07	*Erwinia amylovora* NBRC 12687(T)	98.49	100	OM838449
SRD08	*Microbacterium enclense* NIO-1002	99.1	100	OM838460
SRD06	*Microbacterium hydrothermale* 0704C9-2	99.17	100	OM838458
ST01	*Pseudomonas oryzihabitans* NBRC 102199	97.37	76.8	OM838463
SRD09	*Rhodococcus kroppenstedtii* DSM 44908	100	100	OM838461
AR01	*Rothia halotolerans* YIM 90716	98.97	100	OM838448

Table S2. The closest relatives of endophytic fungal strains isolated from Symptomatic seedlings (30-40 DAS) that tested negative for phytoplasma.

Strain	Closest relative (GenBank ITS region database)	% Similarity	% Query Coverage	GenBank Acc. No.
SWF28	*Aspergillus oryzae* NRRL 447	100.00	95	OM838447
SWF7	*Colletotrichum aenigma* ICMP 18608	99.46	97	OM838428
SWF18	*Colletotrichum aenigma* ICMP 18608	99.59	99	OM838437
SWF2	*Colletotrichum aeschynomenes* ICMP 17673	99.64	99	OM838425
SWF14	*Colletotrichum aeschynomenes* ICMP 17673	99.10	100	OM838433
SWF17	*Colletotrichum aeschynomenes* ICMP 17673	99.46	100	OM838436
SWF21	*Colletotrichum aeschynomenes* ICMP 17673	99.46	100	OM838440
SWF1	*Colletotrichum cobbittiense* BRIP 66219	99.30	99	OM838424
SWF5	*Colletotrichum cobbittiense* BRIP 66219	99.82	99	OM838426
SWF8	*Colletotrichum cobbittiense* BRIP 66219	99.82	99	OM838429
SWF10	*Colletotrichum cobbittiense* BRIP 66219	99.46	100	OM838431
SWF15	*Colletotrichum cobbittiense* BRIP 66219	99.46	99	OM838434
SWF9	*Colletotrichum eriobotryae* GLMP 1935	99.82	99	OM838430
SWF19	*Colletotrichum eriobotryae* GLMP 1935	99.82	99	OM838438
SWF22	*Colletotrichum eriobotryae* GLMP 1935	99.61	99	OM838441
SWF25	*Colletotrichum eriobotryae* GLMP 1935	99.27	98	OM838444
SWF16	*Colletotrichum guajavae* IMI 349845	100.00	99	OM838435
SWF26	*Fusarium cassiae* MFLUCC 18-0573	98.63	100	OM838445
SWF13	*Neofusicoccum vitifusiforme* CBS 110887	98.08	99	OM838432
SWF20	*Neofusicoccum vitifusiforme* CBS 110887	98.20	99	OM838439
SWF23	*Neofusicoccum vitifusiforme* CBS 110887	98.20	99	OM838442
SWF24	*Neofusicoccum vitifusiforme* CBS 110887	98.02	99	OM838443
SWF6	*Neofusicoccum parvum* CMW 9081	100.00	91	OM838427
SWF27	*Neofusicoccum parvum* CMW 9081	100.00	91	OM838446
